# Critical behavior of nonequilibrium depinning transitions for vortices driven by current and vortex density

**DOI:** 10.1038/s41598-022-05504-4

**Published:** 2022-01-28

**Authors:** T. Kaji, S. Maegochi, K. Ienaga, S. Kaneko, S. Okuma

**Affiliations:** Department of Physics, Tokyo Institute of Technology, 2-12-1, Ohokayama, Meguro-ku, Tokyo 152-8551 Japan

**Keywords:** Physics, Condensed-matter physics, Phase transitions and critical phenomena

## Abstract

We study the critical dynamics of vortices associated with dynamic disordering near the depinning transitions driven by dc force (dc current *I*) and vortex density (magnetic field *B*). Independent of the driving parameters, *I* and *B*, we observe the critical behavior of the depinning transitions, not only on the moving side, but also on the pinned side of the transition, which is the first convincing verification of the theoretical prediction. Relaxation times, $\tau (I)$ and $\tau (B)$, to reach either the moving or pinned state, plotted against *I* and *B*, respectively, exhibit a power-law divergence at the depinning thresholds. The critical exponents of both transitions are, within errors, identical to each other, which are in agreement with the values expected for an absorbing phase transition in the two-dimensional directed-percolation universality class. With an increase in *B* under constant *I*, the depinning transition at low *B* is replaced by the *repinning* transition at high *B* in the peak-effect regime. We find a trend that the critical exponents in the peak-effect regime are slightly smaller than those in the low-*B* regime and the theoretical one, which is attributed to the slight difference in the depinning mechanism in the peak-effect regime.

## Introduction

Many-particle systems subjected to an external driving force exhibit a variety of nonequilibrium phases and phase transitions, such as a plastic depinning transition^1–14^ and a reversible-irreversible transition (RIT)^15–26^. The depinning phenomenon of collectively interacting particle systems driven over random substrates has been observed ubiquitously^13^ in various physical systems^6–14,27–29^, including sliding charge density waves^30^, Wigner crystals^31,32^, colloids^3,33^, magnetic domain walls^34^, and superconducting vortices in type-II superconductors^1,2,4,5,35^. Using a superconducting vortex system in amorphous (*a*-)$\hbox {Mo}_{{x}}\hbox {Ge}_{1-x}$ films with random pinning centers, we have previously shown^9,12,20^ that the depinning transition is a nonequilibrium phase transition, as predicted by numerical simulation^5^. In our experimental protocol, we first prepare an ordered initial vortex configuration where many vortices are depinned from random pinning sites or equivalently, an ordered lattice involving a small number of dislocations (topological defects). Then, we apply a small dc current *I* (dc force) with a sharp rise, so that the driven vortices are gradually pinned by random pinning centers and transform into a less organized configuration. This transient process called a dynamic disordering is detected from the time evolution of a voltage *V*(*t*) induced by vortex motion that decays toward a steady-state voltage $V^{\infty }(\equiv V(t\rightarrow \infty ))$, where the voltage corresponds to the average velocity of vortices. The relaxation time $\tau (I)$ for the system to settle into the moving steady state ($V^{\infty }>0$) exhibits a power-law divergence at the depinning current $I_{d}$ with critical exponents $\nu$ = 1.4 ± 0.4^9,12,20^, within error bars, in agreement with the value expected for an absorbing phase transition in the two-dimensional (2D) directed-percolation (DP) universality class^36–38^. Using ac drive^39^, we have also observed the critical behavior of the depinning transition with the critical exponent close to that for the dc drive, further demonstrating the universality of the nonequilibrium depinning transition^5^.

In these experiments, however, the critical behavior of the depinning transition has been observed only on the moving (fluctuating diffusing [active]) side of the transition^9,12,20,39^, although that of RIT has been reported on both sides of the transition^20,24,26^. This is because it is difficult to obtain a reliable data of *V*(*t*) in the pinned (non-fluctuating quiescent [absorbing]) phase, where *V*(*t*) relaxes to $V^{\infty }$ = 0. It was also predicted theoretically that one could hardly observe the nonequilibrium absorbing phase transition, since a perfect non-fluctuating state could not be realized in actual systems, and that even though the fluctuations were strongly suppressed, they could still be strong enough to “soften” the transition, preventing the accurate determination of the critical exponents^36^. The diverging $\tau$ on both sides of the transition has been reported in a vortex system of $\hbox {NbS}_{{2}}$ single crystals^40^. However, the transition is induced by “jamming” of vortices at large dc currents, which is different from the usual depinning that occurs at smaller currents. In fact, the values of $\tau$ reported in^40^ are by approximately five orders of magnitude larger than those in the depinning transition and the extracted critical exponent, $1.6\pm 0.12$, is larger than $\nu =$ 1.4 for the depinning transition, indicating a different universality class from that of the depinning transition and the absorbing phase transition in the 2D DP universality class.

In the ordinary type-II superconductors, the depinning current $I_{d}$ is a function of the magnetic field *B*, and it decreases with an increase in *B* and vanishes at the melting field $B_{m}$ of the vortex solid. This implies that the depinning transition occurs not only when the dc current *I* is increased at constant field *B*, as conducted in previous studies, but also when *B* (i.e., the vortex density) is increased at constant *I*. Within the linear approximation, the critical behavior characterized by the critical exponents is unchanged when *B* is used as a driving parameter instead of *I*. We consider, however, that this is not a trivial issue in real systems. First, the experimental results have shown that the relative width of the critical region of the depinning transition, $|I-I_{c}|/I_{c}$, is very large, typically spanning the range up to $\sim 1$, where $I_{c}$ is the critical current for the depinning transition. This width is much larger than that expected for typical equilibrium critical phenomena^41,42^ and clearly beyond the linear approximation. Second, some experiments studying critical phenomena of phase transitions^43,44^ have shown that the critical exponents of the transition depend on the parameters that drive the transition. For example, critical exponents of the 2D superconductor-insulator transition (SIT) are markedly different depending on whether the SIT is driven by decreasing the film thickness (increasing the normal state resistance) or increasing the magnetic field *B*^43^.

In addition, in the vortex system the presence of the peak effect would make the problem nontrivial and more interesting. For superconductors with moderately strong pinning, as *a*-$\hbox {Mo}_{{x}}\hbox {Ge}_{1-x}$ films studied in this work, $I_{d}(B)$ exhibits a small peak at a certain field $B_{p}$ in the high-*B* region just prior to melting of the vortex lattice. $B_{p}$ marks the structural transition of vortex solids from the ordered lattice or weakly-disordered vortex lattice (Bragg glass) to disordered amorphouslike vortex glass^45,46^. In our *a*-$\hbox {Mo}_{{x}}\hbox {Ge}_{1-x}$ film the weakly disordered vortex lattice (Bragg glass phase) is more likely than the vortex lattice with negligibly small pinning. This peak called a peak effect originates from combined effects of the softening of the vortex lattice before its melting and the random pinning potential due to quenched disorder in the sample. In the particular field region $B\lesssim B_{p}$ in the peak-effect regime, where $I_{d}(B)$ $increases$ with an increase in *B*, a $repinning$ transition from the moving state to pinned state takes place with an increase in *B* (the vortex density) under the fixed current *I* slightly below the depinning current $I_{d}(\equiv I_{d,p})$ at $B_{p}$. This leads to an interesting question of whether the critical behavior of the repinning transition in the high-*B* region $(B\lesssim B_{p})$ is the same as that of the depinning transition in the low-*B* region $(B\ll B_{p})$. Moreover, when we select the specific value of *I*, corresponding to $I_{d,p}$, and *B* is swept near $B_{p}$, the pinned phase appears only at $B_{p}$. Thus, we are able to study the critical behavior of the depinning transition in an unusual situation where the pinned phase is only a point.

In this work, we observe the critical behavior of the depinning transitions, not only on the moving side, but also on the pinned side of the transition, independent of the driving parameters, *I* and *B*. The relaxation times, $\tau (I)$ and $\tau (B)$, to reach either the moving or pinned state, plotted against *I* and *B*, respectively, show a power-law divergence at the depinning thresholds, $I_{c}$ and $B_{c}$. The critical exponents of both transitions are, within errors, identical to each other. In the low-*B* region below the peak effect regime, these exponents are in agreement with the value expected for the absorbing phase transition in the 2D DP class. With an increase in *B* under constant *I*, the depinning transition at low $B (\equiv B_{cL})$ is replaced by the repinning transition at high $B (\equiv B_{cH})$ in the peak-effect regime. While the critical behaviors are similar to each other, we find a trend that the critical exponents in the peak-effect regime are slightly smaller than those in the low-*B* regime and than the theoretical one. Its origin is attributed to the slight difference in the depinning mechanism in the peak-effect regime. We also find that $\tau (B)$ obtained under the particular current $I_{d,p}$ shows a power-law divergence at $B_{p}$, indicating that the critical behavior in the moving phase stays unchanged even when the pinned phase shrinks to a point at $B_{p}$.

## Results and discussion

**Figure 1:**
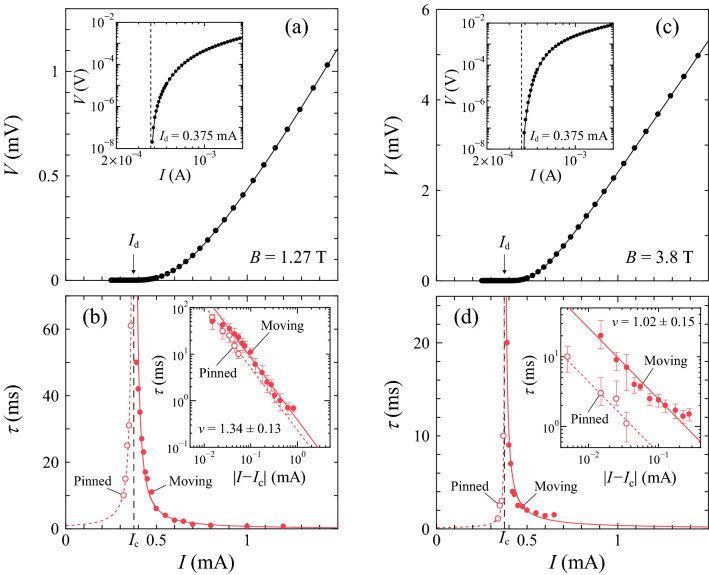
The critical divergence of the relaxation time $\tau (I)$ at the current-driven depinning transition ($I_{c}$). (**a**, **c**) *I*–*V* characteristics at 4.1 K in (**a**) 1.27 T and (**c**) 3.8 T on a linear-linear scale. Inset: *I*–*V* data plotted on a log–log scale. A vertical arrow and dashed line in each main panel and inset, respectively, indicate the depinning current $I_{d}$ = 0.375 mA defined using a $10^{-8}$ V criterion. Other lines are guide to the eye. (**b**, **d**) The critical divergence of $\tau (I)$ at the *I*-driven depinning transition in (**b**) 1.27 T and (**d**) 3.8 T. The open and solid red circles represent $\tau$ for $I\le$ 0.37 mA and $I\ge$ 0.39 mA, respectively, showing a power-law divergence at 0.375 ± 0.005 mA($\equiv I_{c}$) from both sides, as marked with a vertical dashed line. Inset: $\log {\tau }$ versus $\log {|I-I_{c}|}$ in (**b**) 1.27 T and (**d**) 3.8 T, where symbols are the same as in the main panel. Both the dotted and full red lines indicate the power-law fits by $\tau \propto |I-I_{c}|^{-\nu }$ with (**b**) $\nu$ = 1.34 ± 0.13 and (**d**) $\nu$ = 1.02 ± 0.15. The error bars in $\tau$ mainly correspond to the fitting errors resulting from the uncertainty in determining *a*.

In Fig. 1a,c, we show the current-voltage (*I*–*V*) characteristics at 4.1 K in 1.27 and 3.8 T, respectively, on a linear-linear scale. In the insets, the *I*–*V* data in the main panels, including additional data, are plotted on a log-log scale. We define $I_{d}$ as a threshold current at which the vortices start to move, using a $10^{-8}$ V criterion^9^. The location of $I_{d}$ = 0.375 mA for each field is marked with a vertical arrow and dashed line in the main panel and inset, respectively. Upward curvature of the *I*–*V* characteristics just above $I_{d}$ in the main panels indicates that the vortex flow immediately after the depinning is plastic flow, as described later.

**Figure 2:**
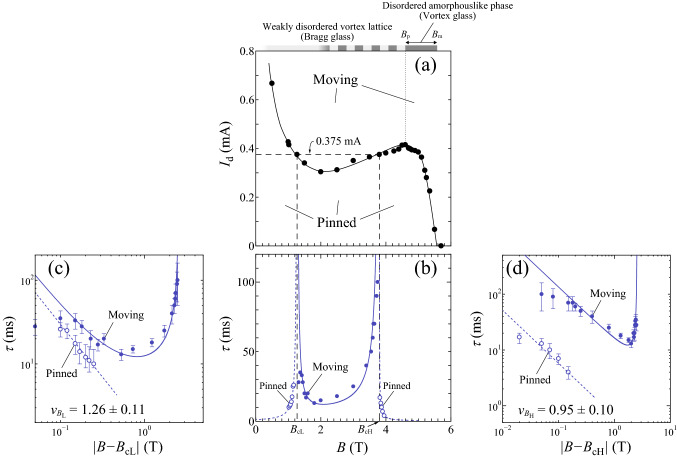
The critical divergence of the relaxation time $\tau (B)$ at the density-driven depinning transition ($B_{cL}$) and repinning transition ($B_{cH}$). (**a**) The *B* dependence of $I_{d}$ at 4.1 K, where a full line is a guide to the eye. $I_{d}(B)$ corresponds to the transition line separating the pinned phase ($<I_{d}(B)$) from the moving phase ($>I_{d}(B)$). $B =$ 1.27 T($\equiv B_{cL}$) and 3.8 T($\equiv B_{cH}$) indicated with vertical dashed lines give the same $I_{d}=$ 0.375 mA marked with a horizontal dashed line. (**b**) $\tau$ measured under constant *I* = 0.375 mA for $B<$1.27 T and $B>$3.8 T (open blue circles) and for 1.27$<B<$3.8 T (solid blue circles) plotted against *B*, showing the power-law divergence on both sides of the two threshold fields; $B_{cL}=$ 1.27 T and $B_{cH}=$ 3.8 T, as indicated with vertical dashed lines. (**c**) $\log {\tau }$ versus $\log {|B-B_{cL}|}$ and (**d**) $\log {\tau }$ versus $\log {|B-B_{cH}|}$, where the symbols are the same as in (**b**). All the data below 3.8 T and above 1.27 T are plotted in (**c**) and (**d**), respectively. The blue dotted line in (**c**) and that for $B<B_{cL}$ in (**b**) represent the power-law fits by $\tau \propto |B-B_{cL}|^{-\nu _{B_{L}}}$ with $\nu _{B_{L}}$ = 1.26 ± 0.11. The blue dotted line in (**d**) and that for $B>B_{cH}$ in (**b**) indicate the power-law fits by $\tau \propto |B-B_{cH}|^{-\nu _{B_{H}}}$ with $\nu _{B_{H}}$ = 0.95 ± 0.10. The blue full lines in (**b**–**d**) are the fits to the sum of the two power-law functions, $\tau = a_{L}|B-B_{cL}|^{-\nu _{B_{L}}} + a_{H}|B-B_{cH}|^{-\nu _{B_{H}}}$, with $\nu _{B_{L}}$ = 1.26 and $\nu _{B_{H}}$ = 0.95, where $a_{L}$ and $a_{H}$ are the fitting parameters of the same order of magnitude. The error bars in $\tau$ mainly correspond to the fitting errors resulting from the uncertainty in determining *a*.

Shown in Fig. 2a is the *B* dependence of $I_{d}$, which is interpreted as a phase diagram of the pinned and moving vortex phases: A full line $I_{d}(B)$ corresponds to the transition line between the pinned phase ($<I_{d}(B)$) and the moving phase ($>I_{d}(B)$). The peak effect is clearly observed in the field range $B=$ 2–5.6 T$(=B_{m})$, where the peak field $B_{p} =$4.6 T marks the structural transition from the Bragg glass to the vortex glass at equilibrium. Because of the non-monotonic dependence of $I_{d}$ on *B*, three fields, such as $B =$1.27 T($\equiv B_{cL}$), 3.8 T($\equiv B_{cH}$), and 5.24 T, give the same $I_{d} =$0.375 mA.

**Figure 3:**
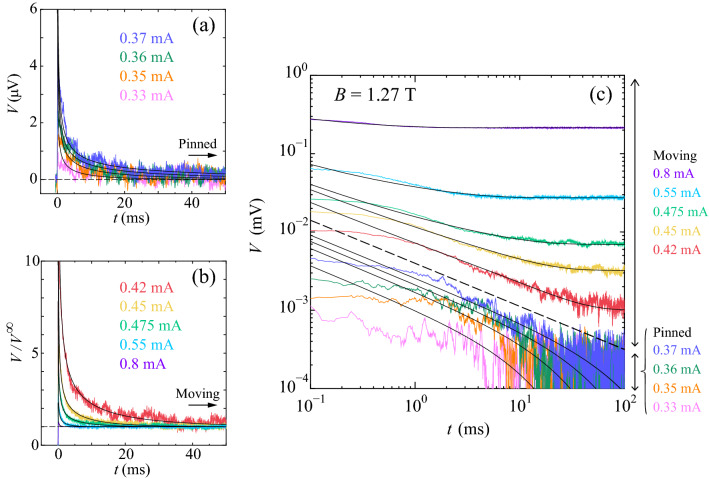
The dynamic disordering of vortices driven by dc currents around the depinning transition ($I_{c}=$ 0.375 mA). (**a**) *V*(*t*) and (**b**) $V(t)/V^{\infty }$ at 4.1 K in 1.27 T just after the dc currents *I* below and above $I_{d}(=$ 0.375 mA), respectively, were suddenly applied to the initial vortex assemblies at $t=0$: (**a**) $I=$ 0.33, 0.35, 0.36, and 0.37 mA from bottom to top and (**b**) $I=$ 0.42, 0.45, 0.475, 0.55, and 0.80 mA from top to bottom. Horizontal dashed lines in (**a**) and (**b**) mark the steady-state values of $V(t)=$ 0 (pinned phase) and $V(t)/V^{\infty }=$ 1 (moving phase), respectively. (**c**) Replots of the data shown in (**a**) and (**b**) as $\log {V}$ versus $\log {t}$: $I=$ 0.33, 0.35, 0.36, 0.37, 0.42, 0.45, 0.475, 0.55, and 0.8 mA from bottom to top. The dashed line represents the slope of $-a=-0.55\pm 0.1$, within errors, nearly consistent with the theoretical value of $a\approx$ 0.45 for the DP universality class in 2D or $a\approx$ 0.5 for the conserved DP universality class in 2D^36,37^. Full lines in (**a**–**c**) indicate the fits to Eq. (1).

Figure 3a,b, respectively, show the time-dependent voltage, *V*(*t*) and $V(t)/V^{\infty }$, at 4.1 K in 1.27 T just after the dc currents, $I=$ 0.33, 0.35, 0.36, and 0.37 mA from bottom to top and $I=$ 0.42, 0.45, 0.475, 0.55, and 0.80 mA from top to bottom, below and above $I_{d}(=$ 0.375 mA) were suddenly applied to the initial vortex assemblies at $t=0$. Here, the ordered initial vortex configuration was prepared by shaking the vortices, using an ac current with a frequency of 10 kHz and an amplitude yielding an ac voltage with an amplitude of 0.1 mV^12^. We commonly observed a decay of *V*(*t*) toward a final steady state, indicative of dynamic disordering. This behavior is essentially the same as that observed in the similar vortex system^9,20^ and originally found in $\hbox {NbSe}_{{2}}$ single crystals^2^. It has been suggested previously that information on dynamic disordering^7^ and the depinning transition^47^ may be involved in individual voltage pulses in response to the ac drive with a rectangular pulse shape as well as in *V*(*t*) in response to the dc drive. It is seen from Fig. 3a that the relaxation is longer for larger *I*, while Fig. 3b shows the longer relaxation for smaller *I*, indicating that a peak in the relaxation time $\tau (I)$ occurs at around $I=$ 0.37–0.42 mA.

To extract $\tau$ for the system to reach the steady state, we fit *V*(*t*) to the following relaxation function presented in^5,17^:

1 $$\begin{aligned} V(t)=(V^{0}-V^{\infty })exp{(-t/\tau )}/t^a + V^{\infty }. \end{aligned}$$

Here $V^{0}$ and $V^{\infty }$ are the initial and steady-state voltages, respectively, and $\tau$ is the characteristic time at which the relaxation crosses over from a power-law decay with an exponent *a* to an exponential decay, as shown in Fig. 3c. Hence, *a* is relevant very close to the transition where $\tau \rightarrow \infty$^5,13,17^. In Fig. 3c, we replot all the data shown in Fig. 3a,b as $\log {V}$-$\log {t}$ plots. We find that as *I* approaches closer to 0.37-0.42 mA, the replotted data fall on nearly a straight line with a slope of $-a=-0.55$, as indicated with a dashed line. The clear and systematic discrepancy between the fitting lines and the experimental curves at $t<$ 1 ms is inevitable from the functional form of Eq. (1). To extract the values of $\tau$, the fitting in the larger *t* regime where the relaxation crosses over from a power-law decay to an exponential decay is important and the deviation from the fitting at smaller $t(<$ 1 ms) does not affect the extracted values of $\tau$. The obtained value of $a=0.55\pm 0.1$ is almost consistent with the DP theory^36,37^, which predicts that the fraction of active (moving) particles, in the case of the depinning transition, obeys the power-law time dependence at the critical point with an exponent $a\approx 0.45$ for the DP class or $a\approx 0.5$ for the conserved DP class.

The full lines in Fig. 3a,b,c display the results of the fits to Eq. (1) using $a=0.55$. In Fig. 1b, the values of $\tau$ thus obtained are plotted as a function of *I* with open and solid red circles for $I\le$0.37 mA and $I\ge$0.39 mA, respectively. They show a power-law divergence at 0.375 ± 0.005 mA($\equiv I_{c}$) from both sides, as marked with a vertical dashed line. As far as we know, this is the first experimental observation of the critical divergence of $\tau$ in the pinned phase ($I<I_{c}$) of the depinning transition. The inset of Fig. 1b displays the plots of all the values of $\tau$ against $|I-I_{c}|$ on a double logarithmic scale, i.e., $\log {\tau }$ versus $\log {|I-I_{c}|}$, where symbols are the same as in the main panel. Both the dotted and full red lines in the main panel and inset indicate the power-law fits by $\tau \propto |I-I_{c}|^{-\nu }$ with $\nu$=1.34 ± 0.13, which is, within error bars, in agreement with the theoretical value of $\nu =1.295\pm 0.006$ expected for the absorbing phase transition in the DP universality class in 2D^36^ and $\nu =1.225\pm 0.029$ for the conserved 2D DP class.

In 3.8 T slightly lower than $B_{p}=$ 4.6 T in the peak-effect regime, where the pinning is very effective, we also measured the transient voltage *V*(*t*) just after the dc currents *I* below and above $I_{d}=$ 0.375 mA were suddenly applied to the initial vortex assembly with a relatively ordered configuration, which was prepared by the same ac drive as in 1.27 T. We again observed the decay of *V*(*t*) to the steady-state voltage $V^{\infty }$. The relaxation time $\tau$ was extracted from *V*(*t*), using the same analysis as in 1.27 T. The main panel and inset of Fig. 1d display the $\tau$ versus *I* and $\log {\tau }$ versus $\log {|I-I_{c}|}$ plots, respectively, where the open and solid red circles correspond to $\tau$ measured at $I\le$0.37 mA and $I\ge$0.39 mA, respectively. The values of $\tau (I)$ again show a power-law divergence at 0.375 ± 0.005 mA($\equiv I_{c}$) from both sides, as indicated with a vertical dashed line. Both the dotted and full red lines in the main panel and inset indicate the power-law fits by $\tau \propto |I-I_{c}|^{-\nu }$ with $\nu$ = 1.02 ± 0.15, which is slightly smaller than $\nu$ = 1.34 ± 0.13 in 1.27 T.

**Figure 4:**
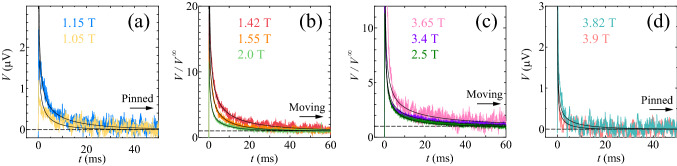
The dynamic disordering of vortices driven by a dc current around the density-driven depinning transition ($B_{cL}=$ 1.27 T) and repinning transition ($B_{cH}=$ 3.8 T). (**a**–**d**) Voltage responses for the relatively ordered initial vortex configuration subjected to the dc current *I* = 0.375 mA at 4.1 K in different *B*: (**a**) *V*(*t*) in $B=$ 1.05 and 1.15 T from bottom to top, (**b**) $V(t)/V^{\infty }$ in $B=$ 1.42, 1.55, and 2.0 T from top to bottom, (**c**) $V(t)/V^{\infty }$ in $B=$ 2.5, 3.4, and 3.65 T from bottom to top, and (**d**) *V*(*t*) in $B=$ 3.82 and 3.9 T from top to bottom. The horizontal dashed lines mark the steady-state values of (**a**) $V(t)=0$ (pinned phase), (**b**) $V(t)/V^{\infty }=$ 1 (moving phase), (**c**) $V(t)/V^{\infty }=$ 1 (moving phase), and (**d**) $V(t)=$ 0 (pinned phase). Full lines in (**a**–**d**) indicate the fits to Eq. (1).

Next, we focus on the critical behavior of the depinning transition that is driven by increasing *B* in the low-*B* region $(B\ll B_{p})$ and by decreasing *B* in the high-*B* region $(B\lesssim B_{p})$ under the constant current $I=$ 0.375 mA slightly lower than $I_{d,p}=$ 0.415 mA (i.e., the depinning current for $B_{p}$) at 4.1 K. Here, the experiment was performed by keeping the field constant and then switching on the current $I =$ 0.375 mA suddenly. Shown in Fig. 4a–d are the voltage responses *V*(*t*) for the relatively ordered initial vortex configuration, which was prepared by the same ac drive as mentioned above, subjected to the dc current *I* = 0.375 mA in different *B*: Fig. 4a shows *V*(*t*) in $B=$ 1.05 and 1.15 T from bottom to top, while Fig. 4b displays $V(t)/V^{\infty }$ in $B=$ 1.42, 1.55, and 2.0 T from top to bottom. Figure 4c shows $V(t)/V^{\infty }$ in $B=$ 2.5, 3.4, and 3.65 T from bottom to top, while Fig. 4d displays *V*(*t*) in $B=$ 3.82 and 3.9 T from top to bottom. It is commonly observed that *V*(*t*) decays to the steady-state voltage $V^{\infty }$. All the data of *V*(*t*) in Fig. 4a,d show $V^{\infty }=0$, indicative of the pinned phase, whereas those in Fig. 4b,c exhibit $V^{\infty }>0$, reflecting the moving phase. Full lines in Fig. 4a–d represent the results of the fits to Eq. (1).

From *V*(*t*), we again extract the relaxation time $\tau$ for various *B*. In Fig. 2b, we plot all the values of $\tau$ measured under constant *I* = 0.375 mA for $B<$1.27 T and $B>$3.8 T with open blue circles and for 1.27$<B<$3.8 T with solid blue circles. The power-law divergence of $\tau$ is clearly observed on both sides of the two threshold fields; i.e., $B_{cL} =$1.27 T and $B_{cH} =$ 3.8 T, as indicated with vertical dashed lines. Shown in Fig. 2c,d are the plots of $\tau$ against $|B-B_{cL}|$ and $|B-B_{cH}|$ on a double logarithmic scale, i.e., $\log {\tau }$ versus $\log {|B-B_{cL}|}$ and $\log {\tau }$ versus $\log {|B-B_{cH}|}$, respectively, where the symbols are the same as in Fig. 2b. The blue dotted line in Fig. 2c and that for $B<B_{cL}$ in Fig. 2b represent the power-law fits by $\tau \propto |B-B_{cL}|^{-\nu _{B_{L}}}$ with $\nu _{B_{L}}$ = 1.26 ± 0.11. The blue dotted line in Fig. 2d and that for $B>B_{cH}$ in Fig. 2b indicate the power-law fits by $\tau \propto |B-B_{cH}|^{-\nu _{B_{H}}}$ with $\nu _{B_{H}}$ = 0.95 ± 0.10. The blue full lines in Fig. 2b,c,d are the fits to the sum of the two power-law functions, $\tau = a_{L}|B-B_{cL}|^{-\nu _{B_{L}}} + a_{H}|B-B_{cH}|^{-\nu _{B_{H}}}$, with $\nu _{B_{L}}$ = 1.26 and $\nu _{B_{H}}$ = 0.95, where $a_{L}$ and $a_{H}$ are the fitting parameters of the same order of magnitude. We find that the critical behaviors of the depinning and repinning transitions at $B_{cL}$ and $B_{cH}$ driven by *B* (vortex density) for fixed $I=$ 0.375 mA are similar to those of the depinning transitions driven by *I* (driving force) for fixed $B=B_{cL}$ and $B_{cH}$, respectively. Specifically, the values of the critical exponent $\nu _{B}$, i.e., $\nu _{B_{L}}$ = 1.26 ± 0.11 and $\nu _{B_{H}}$ = 0.95 ± 0.10, for the *B*-driven transitions at $B_{cL}$ and $B_{cH}$ are, within error bars, in agreement with $\nu$ = 1.34 ± 0.13 and $\nu$ = 1.02 ± 0.15 for the *I*-driven transitions in $B_{cL}$ and $B_{cH}$, respectively. The results indicate that the critical exponents for $\tau$ are nearly independent of the parameters, *I* and *B*, that drive the transition.

One can notice the asymmetry in the $\tau$ versus *I* plot about $I_{c}(=$ 0.375 mA) under 3.8 T in Fig. 1d and in the $\tau$ versus *B* plot about $B_{cH}(=$ 3.8 T) under 0.375 mA in Fig. 2b. The asymmetry arises from a smaller relaxation time $\tau$ in the pinned phase than in the moving phase. As mentioned later, this trend is commonly observed in the peak-effect regime. In particular, in the amorphouslike vortex glass phase ($B>B_{p}$) we can no longer detect the clear relaxation ($\tau \sim 0$). The smaller relaxation time is partly attributed to the fact that the initial vortex configuration prepared by the ac drive is not ordered enough. However, we are unable to explain comprehensively why the fast relaxation mechanism is present only in the pinned phase of the peak-effect regime. In the vicinity of $B_{p}$, an emergence of the metastability with respect to vortex configurations may play a role. This is an interesting problem to be solved in future research. It is also of interest to note that the asymmetry in $\tau$ about the transition is observed in different physical systems that exhibit RIT^17,26^, which would fall into the same universal class as the depinning transition and the absorbing transition in DP.

**Figure 5:**
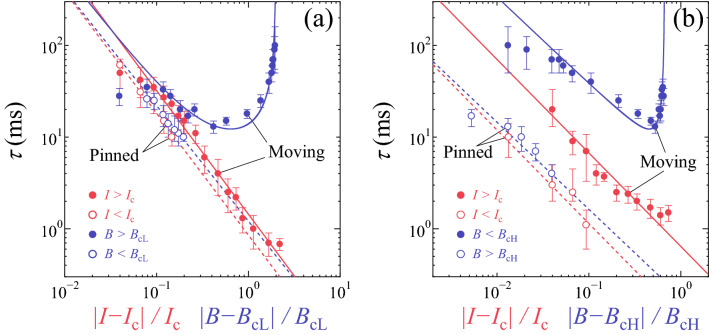
The relative width of the critical region. The quantities for the abscissa indicate the normalized distance from the critical point. (**a**) $\log {\tau }$ measured in $B=$ 1.27 T plotted against $|I-I_{c}|/I_{c}$ (red circles) and that at $I=$ 0.375 mA against $|B-B_{cL}|/B_{cL}$ (blue circles), which are collected from the data in Figs 1b and 2c, respectively. All the symbols and lines correspond to those in Figs. 1b and 2c. (**b**) $\log {\tau }$ measured in 3.8 T plotted against $|I-I_{c}|/I_{c}$ (red circles) and that at 0.375 mA against $|B-B_{cH}|/B_{cH}$ (blue circles), which are collected from the data in Figs. 1d and 2d, respectively. All the symbols and lines are the same as those in Figs. 1d and 2d. The open and solid circles in (**a**, **b**) correspond to the data in the pinned and moving phases, respectively. The error bars in $\tau$ mainly correspond to the fitting errors resulting from the uncertainty in determining *a*.

To examine the extension of the critical regions with respect to the driving parameters, *I* and *B*, comparatively, in Fig. 5a, we plot with red and blue circles, respectively, $\log {\tau }$ measured in $B=$ 1.27 T($=B_{cL}$) against $|I-I_{c}|/I_{c}$ and $\log {\tau }$ measured at $I=$ 0.375 mA($=I_{c}$) against $|B-B_{cL}|/B_{cL}$, which are collected from the data in Figs. 1b and 2c. All the symbols and lines correspond to those in Figs. 1b and 2c. Similarly, in Fig. 5b, we plot with red and blue circles $\log {\tau }$ in 3.8 T($=B_{cH}$) against $|I-I_{c}|/I_{c}$ and $\log {\tau }$ at 0.375 mA($=I_{c}$) against $|B-B_{cH}|/B_{cH}$, which are collected from the data in Figs. 1d and 2d, respectively. All the symbols and lines are the same as those in Figs. 1d and 2d. The open and solid circles in Fig. 5a,b correspond to the data in the pinned and moving phases, respectively. It is found that the relative width of the critical region, $|I-I_{c}|/I_{c}$, $|B-B_{cL}|/B_{cL}$, and $|B-B_{cH}|/B_{cH}$, spans the broad range up to near 1: It ranges from (4–7)$\times 10^{-2}$ to (0.7–2)$\times 10^{0}$ and from (1–4)$\times 10^{-2}$ to (1–2)$\times 10^{-1}$ in the moving phase and pinned phases, respectively, which are much larger than that of typical equilibrium critical phenomena^41,42^. Note the trivial fact that in the pinned phase, the relative width does not exceed 1, which is given when $I=B=$ 0. In general, in the vicinity of the phase transition where the linear approximation is valid, the critical exponents of the transition are independent of the parameters that drive the transition. The observed independence of the critical exponents from the driving parameters is somewhat surprising, considering that the critical regions observed here are very large, clearly going beyond the linear approximation. The similar large critical region has been reported in the nonequilibrium RIT in various systems^17,22,25^, including the vortex system^24^, where the parameter-independent critical exponents with $\nu =$ 1.3–1.4 have been found for the shear-driven and density-driven transitions^26^.

Some previous experiments showed that the critical exponents are significantly dependent on the driving parameters when the underlying mechanisms driving the transition are fundamentally different^44^. Our results of $\nu \approx \nu _{B}$ indicate that the driving mechanisms of *I* and *B* are essentially the same or not different enough to change significantly the universality class of the transition. However, we focus on a trend that $\nu \approx \nu _{B_{H}}\approx$1.0 for $B=$ 3.8 T $(\lesssim B_{p})$ obtained in the peak-effect regime is slightly smaller than $\nu \approx \nu _{B_{L}}\approx$1.3 for $B=$ 1.27 T obtained below the peak-effect regime and than $\nu =$ 1.295 (or 1.23) predicted by the 2D DP (or conserved DP) theory. This trend was also observed in our previous work, although the critical exponents were derived based only on the data in the moving phase^12^. While the exact reason remains elusive, it might be in part due to insufficient experimental accuracy of determination of the exponents in the peak-effect regime. Since pinning is effective in the peak-effect regime, as detailed below, the initial vortex configuration prepared by the ac drive is assumed to be not ordered enough for the dynamic disordering to be clearly observed. In fact, in the high-*B* region above $B_{p}$, corresponding to the highly disordered amorphouslike vortex-glass phase, we were not able to obtain reliable data of *V*(*t*) showing the dynamic disordering in the pinned phase even in the vicinity of $I_{d}$.

We now consider from the present data that the observed trend is intrinsic and it may originate from the slight difference in the depinning mechanism in the peak-effect regime. Below the peak-effect regime, the vortices form an ordered triangular lattice or weakly disordered lattice called a Bragg glass, which interact with randomly distributed pinning centers. For the relatively stiff lattice, the energy cost to deform the vortex lattice is larger than the energy gain obtained from the vortices being pinned to the random pinning centers^13^. Therefore, a large fraction of the pinning centers remains unoccupied and pinning is ineffective. As the field *B* is increased and an intervortex length $a_{0}(\approx \sqrt{\Phi _{0}/B})$ decreases, the stiffness of the lattice increases, so that the pinning becomes more ineffective. This explains the observed decrease in $I_{d}$ with an increase in *B* and, equivalently, the observed depinning phenomenon caused by an increased *B* at constant *I*. In the peak-effect regime, by contrast, as the field is increased and the liquid phase is approached, the vortex lattice becomes softened and can deform easily. As a result, many of the pinning centers are occupied by vortices and the pinning becomes effective. This is why $I_{d}$ increases with an increase in *B* and the repinning(/depinning) occurs with an increase(/decrease) in *B* at constant $I (<I_{d,p})$. As *B* is increased up to $B_{p}$, the whole vortex system changes to the vortex glass. In the vicinity of $B_{p}$, the vortex dynamics becomes complex because an energy landscape with different vortex configurations with similar free energy, separated by energy barriers, emerges^14^. Indeed, we have found earlier from measurements of flow noise in the same vortex system of *a*-$\hbox {Mo}_{{x}}\hbox {Ge}_{1-x}$ films that flow noise shows a sharp rise at $B_{p}$, indicating that the vortex dynamics is most complicated in the vicinity of the order-disorder transition at $B_{p}$^46^.

Intuitively speaking, what we see in the low-*B* region is the depinning of the relatively stiff lattice that weakly couples to the random substrate, whereas in the peak-effect regime we see the depinning of the softer lattice that strongly couples to the random substrate^13^. In superconductors with a moderately strong random pinning potential, such as the *a*-$\hbox {Mo}_{{x}}\hbox {Ge}_{1-x}$ film, local variations in the pinning strength play an important role. When the current *I* close to $I_{d}$ is applied to the vortex lattice, large plastic deformation is produced. As *I* exceeds $I_{d}$, the vortex system enters a state where the chains or rivers of the vortices begin to move with different average velocities, thus exhibiting a plastic flow^13^. In our *a*-$\hbox {Mo}_{{x}}\hbox {Ge}_{1-x}$ film, the plastic depinning and plastic flow are realized in the entire *B* region studied rather than the elastic depinning and elastic flow expected in the weak pinning limit. The plastic flow is confirmed by the *I*–*V* characteristics with positive curvature at $I\gtrsim I_{d}$, as shown in the main panels of Fig. 1a,c.

**Figure 6:**
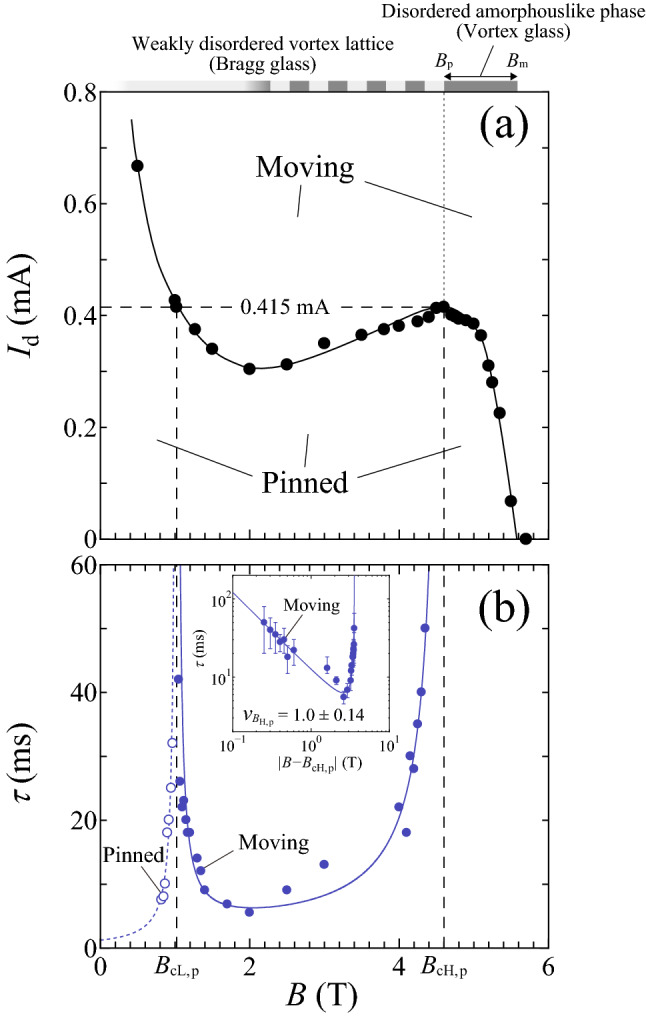
The critical divergence of the relaxation time $\tau (B)$ at the density-driven depinning transition ($B_{cL,p}$) and repinning transition ($B_{cH,p}$). In the high-*B* region, the pinned phase corresponds to a point at $B_{p}$. (**a**) The *B* dependence of $I_{d}$ at 4.1 K, which is the same data as shown in Fig. 2a. A horizontal dashed line marks $I_{d,p}=$ 0.415 mA and a full line is a guide to the eye. (**b**) $\tau$ obtained from *V*(*t*) measured under the fixed $I_{d,p}=$ 0.415 mA plotted against *B*, where open and solid blue circles correspond to the data in the pinned and moving phases, respectively. $\tau$ exhibits a power-law divergence at the peak field $B_{p}=4.6$ T($\equiv B_{cH,p}$) as well as at 1.02 T($\equiv B_{cL,p}$), as indicated with vertical dashed lines in (**a**, **b**). The inset shows $\log {\tau }$ versus $\log {|B-B_{cH,p}|}$. The blue dotted line in the main panel represents the power-law fit by $\tau \propto |B-B_{cL,p}|^{-\nu _{B_{L,p}}}$ with $\nu _{B_{L,p}}$=1.2 ± 0.16. The blue full lines in the main panel and inset are the fit to $\tau = a_{L,p}|B-B_{cL,p}|^{-\nu _{B_{L,p}}} + a_{H,p}|B-B_{cH,p}|^{-\nu _{B_{H,p}}}$ with $\nu _{B_{L,p}}$ = 1.26 and $\nu _{B_{H,p}}$ = 1.0 ± 0.14, where $a_{L,p}$ and $a_{H,p}$ are the fitting parameters of the same order of magnitude. The error bars in $\tau$ mainly correspond to the fitting errors resulting from the uncertainty in determining *a*.

In general, crystalline materials exhibit a plastic behavior where a well-defined number of topological defects are present^13^. A critical behavior appears near yield that is similar to the behavior observed near depinning^48^. This crystal plasticity is different from the plasticity in amorphous systems where topological defects are not well defined. It is not evident whether a similar difference is present between plastic depinning that has properties similar to those associated with crystal plasticity and depinning that is amorphous in nature^13^. The vortex system formed in the peak-effect regime at $B=$ 3.8 T is not a complete amorphouslike vortex glass but a softer vortex lattice composed of the weakly pinned ordered lattice and strongly pinned amorphouslike vortex glass^46^. The results obtained in this work suggest that the critical behaviors of the depinning transitions in the low-*B* and peak-effect regimes are fundamentally the same, nearly independent of whether the depinning is associated with the crystal plasticity or the plasticity of amorphous systems. However, the subtle difference in the critical behavior originating from the difference in the plasticity may be reflected in slightly smaller values of the exponents ($\nu \approx \nu _{B_{H}}\approx$1) in the peak-effect regime, where the crystal plasticity is partially replaced by the plasticity of the amorphous system. In the ac driven dynamic reorganization experiment using the linear ac susceptibility^49^, clear deviations of the critical exponent from a constant value were observed at high frequencies. This observation was interpreted in terms of a possible change in the universality class of the nonequilibrium phase transition.

Finally, we examine whether the same critical behavior of the repinning transition is observed in an unusual situation where the pinned phase shrinks to a point at $B_{p}$. Thus, we have measured *V*(*t*) for the relatively ordered initial vortex configuration subjected to the dc current $I_{d,p}=$ 0.415 mA in different *B*. In Fig. 6b, we plot $\tau$ extracted from *V*(*t*) as a function of *B*, where open and solid blue circles correspond to the data in the pinned and moving phases, respectively. As mentioned above, we were not able to detect reliable signals of *V*(*t*) for $B>B_{p}$ even in a moving phase. The *B* dependence of $I_{d}$ shown in Fig. 2a is also plotted in Fig. 6a, where a horizontal dashed line marks the location of $I_{d,p}=$ 0.415 mA. The power-law divergence of $\tau$ is again visible at the peak field $B_{p} = 4.6$ T($\equiv B_{cH,p}$) as well as at 1.02 T($\equiv B_{cL,p}$), as indicated with vertical dashed lines. The inset of Fig. 6b displays the log-log plots of $\tau$ versus $|B-B_{cH,p}|$. The blue dotted line in the main panel of Fig. 6b represents the power-law fit by $\tau \propto |B-B_{cL,p}|^{-\nu _{B_{L,p}}}$ with $\nu _{B_{L,p}}$ = 1.2 ± 0.16. The blue full lines in the main panel and inset are the fit to the sum of the two power-law functions, $\tau = a_{L,p}|B-B_{cL,p}|^{-\nu _{B_{L,p}}} + a_{H,p}|B-B_{cH,p}|^{-\nu _{B_{H,p}}}$, with $\nu _{B_{L,p}}$ = 1.26 $\pm 0.16$ and $\nu _{B_{H,p}}$ = 1.0 $\pm 0.14$, where $a_{L,p}$ and $a_{H,p}$ are the fitting parameters of the same order of magnitude. Again, we obtain a smaller value of $\nu _{B_{H,p}}\approx$1.0 in the peak-effect regime than $\nu _{B_{L,p}}\approx$1.3 in the low-*B* regime. The results clearly show that the critical behavior on the moving side of the repinning transition remains unchanged even when the pinned phase shrinks to a point at $B_{p}$, corresponding to the order-disorder transition of vortex matter.

## Conclusions

We study the critical dynamics of vortices associated with dynamic disordering near the depinning transitions driven by dc force (*I*) at fixed vortex density (*B*) and driven by vortex density (*B*) at fixed dc force (*I*). Independent of the driving parameters, we observe the critical behavior of the depinning transitions, not only on the moving side, but also on the pinned side of the transition, which is the first convincing experimental verification of the theoretical prediction^5^. The relaxation times, $\tau (I)$ and $\tau (B)$, to reach either the moving or pinned state exhibit a power-law divergence at the depinning thresholds. The critical exponents of the *I*-driven and *B*-driven transitions, $\nu$ and $\nu _{B}$, respectively, are, within error bars, identical to each other, which are in agreement with the value expected for the absorbing phase transition in the 2D DP universality class.

With an increase in *B* under constant *I*, the depinning transition at low field ($B_{cL}$) is replaced by the repinning transition at high field ($B_{cH}$) in the peak-effect regime, whose critical behaviors are similar to each other. The relative width of the critical region, $(I-I_{c})/I_{c}$, $(B-B_{cL})/B_{cL}$, and $(B-B_{cH})/B_{cH}$, which spans the broad range up to near 1, is also nearly independent of the driving parameters. The feature is very similar to that observed recently in the shear-driven and density-driven RIT in the cyclically sheared vortices^26^. However, we find a trend that the critical exponents $\nu \approx \nu _{B_{H}}\approx$1.0 in the peak-effect regime are slightly smaller than $\nu \approx \nu _{B_{L}}\approx$1.3 in the low-*B* region and than $\nu \approx 1.3$ expected for the 2D DP class, whose origin is attributed to the slight difference in the depinning mechanism in the peak-effect regime. Finally, we also find that $\tau (B)$ obtained under the particular current $I_{d,p}(\equiv I_{d}(B_{p}))$ shows a power-law divergence at $B_{p}$, indicating that the critical behavior in the moving phase stays unchanged even when the pinned phase shrinks to a point at $B_{p}$.

We expect that this work will stimulate further studies on the nonequilibrium depinning transition in various systems in which the pinning-depinning phenomenon is observed, such as, sliding charge density waves^27,30^, Wigner crystals^31,32^, colloids^3,33^, magnetic domain walls^34^, skyrmions^28^, friction^27^ and yielding^21,22,25,48^ of solids, superfluid vortices in neutron stars^29^, as well as superconducting vortices in type-II superconductors^1,2,4,5,27,35^.

## Methods

The *a*-$\hbox {Mo}_{{x}}\hbox {Ge}_{1-x}$ film with thickness of 0.35 $\mu$m was prepared by RF sputtering deposition onto a Si substrate mounted on a water cooled copper stage that rotates at 240 rpm^12,20,23,46^. The superconducting transition temperature at which the linear resistivity falls to zero is 6.7 K in zero field. The magnetic field *B* was directed perpendicular to the plane of the film. By applying a current *I*, the vortices move in the direction parallel to the film width of 0.3 mm. The voltage *V* induced by vortex motion was measured with a standard four-probe method, using voltage probes separated at 1.2 mm. We measured the time-evolution of voltage *V*(*t*) immediately after the dc current *I* with a sharp rise ($\lesssim$15 ns) was suddenly applied to the vortex system. The voltage *V*(*t*) enhanced with a preamplifier was acquired and analyzed using a fast-Fourier transform spectrum analyzer with a time-resolution of up to 40 kHz. The film was directly immersed into the liquid $^{4}$He and all the data were taken at 4.1 K. The characteristic length scales for the vortex core and vortex-vortex interaction are the superconducting coherence length and the London penetration length, respectively, which are of the order of $\approx$ $1 \times 10$ and $\approx 5\times 10^{2}$ nm^50,51^. The mean intervortex spacing $a_{0}\approx \sqrt{\Phi _{0}/B}$ was varied from 69 to 20 nm by changing *B* from 0.5 to 5.7 T, respectively, where $\Phi _{0}$ is a flux quantum. In this paper, the dimensionality is related to or corresponds to that of the particle motion in many-particle systems. Thus, the dimensionality of the vortex system is basically 2D, as treated by 2D simulations^1,13,18^. This is supported by the experimental fact that the thickness of the film is comparable to or smaller than the magnetic penetration depth and the possible bending distortions of vortex lines that may cause the deviation from the 2D particles picture can be ignored^24,26^.

## Data Availability

The data that support the findings of this study are available from the corresponding author upon a reasonable request.
